# Development and validation of two bioanalysis methods for the determination of etimicin in human serum and urine by liquid chromatography-tandem mass spectrometry: Applications to a human pharmacokinetic and breakpoint study

**DOI:** 10.3389/fphar.2023.1076046

**Published:** 2023-01-13

**Authors:** Xinge Cui, Xin Zheng, Jianwei Ren, Hongzhong Liu, Yuan Jia, Aiguo Wu, Xiaohong Han

**Affiliations:** ^1^ Clinical Pharmacology Research Center, State Key Laboratory of Complex Severe and Rare Diseases, NMPA Key Laboratory for Clinical Research and Evaluation of Drug, Beijing Key Laboratory of Clinical PK and PD Investigation for Innovative Drugs, Peking Union Medical College Hospital, Chinese Academy of Medical Sciences and Peking Union Medical College, Beijing, China; ^2^ Jiangxi Jemincare Group Co., Ltd., Shanghai, China

**Keywords:** etimicin, LC-MS/MS, human serum and urine, method validation, human pharmacokinetics, aminoglycoside antibiotic

## Abstract

Etimicin is a fourth-generation aminoglycoside antibiotic. It has potent activity and low toxicity when employed for the treatment of Gram-negative and Gram-positive bacterial infections. The pharmacokinetics of etimicin in humans have not been elucidated completely. Two liquid chromatography-tandem mass spectrometry (LC-MS/MS) bioanalytical methods, without the use of any ion-pairing reagents, were developed and validated for the quantification of etimicin in human samples of serum and urine. Using a deuterated reagent as the internal standard, analytes in serum and urine samples were extracted by protein precipitation and dilution before LC-MS/MS analysis, respectively. For the two methods, chromatographic separations were undertaken under isocratic elution of water–ammonia solution–acetic acid (96:3.6:0.2, *v/v/v*) and methanol at 50%:50% and a flow rate of 0.35 ml/min within 5 min. A Waters XTerra MS C18 column (2.1 × 150 mm, 3.5 μm) and a column temperature of 40°C were chosen. A Sciex Qtrap 5500 mass spectrometer equipped with an electrospray ion source was used in both methods under multiple-reaction monitoring in positive-ion mode. The two methods showed good linearity, accuracy, and precision with high recovery and a minimal matrix effect in the range of 50.0–20000 ng/ml for serum samples and 50.0–10000 ng/ml for urine samples, respectively. Carry-over effects were not observed. Etimicin remained stable in human samples of serum or urine under the storage, preparation, and analytical conditions of the two methods. These two simple and reliable methods were applied successfully to a dose-escalation, phase I clinical trial of etimicin in Chinese healthy volunteers after intravenous administration of single and multiple doses. Based on these two methods we ascertained, for the first time, the comprehensive pharmacokinetics of etimicin in humans, which will be used for the exploration of the breakpoint research further.

## 1 Introduction

Etimicin is a fourth-generation aminoglycoside antibiotic developed in China. Etimicin is a modified version of gentamicin C1a (Wang et al., 2011). The antibacterial activity of etimicin is greater or similar to that of other aminoglycosides, but it is less toxic (Zhuang & Cao, 2013). The efficacy of etimicin against Gram-negative and Gram-positive bacterial infections (including those resistant to other aminoglycosides) has been demonstrated (Chaudhary et al., 2012). Etimicin has been employed extensively to treat infections, pneumonia, and urinary-tract infections because of its strong antibacterial activity, limited cross-resistance, good efficacy, and low toxicity (Zhao et al., 2000; Payasi et al., 2010; Huang et al., 2019). Nevertheless, etimicin use can lead to nephrotoxicity, so one must consider if dose adjustment is needed for patients with renal dysfunction (Yao et al., 2020; Shao et al., 2021).

Meanwhile, with the increasing prevalence of bacterial resistance, one must reevaluate if the recommended dose is suitable for antibiotics. The pharmacokinetics of etimicin has been reported only for a single dose of 150 mg (Ouyang, 2007) and 200 mg (Zhang and Yin, 1999; Yin et al., 2000; Wang et al., 2002), and the etimicin concentration was measured by micro-bioassays. Information regarding the pharmacokinetics and clinical breakpoint of etimicin is limited, which hinders the use of etimicin in personalized medicine. To achieve the latter, a comprehensive analysis of the pharmacokinetics in humans must be undertaken. Such studies require rapid analysis of the exact concentration of etimicin in human samples of serum and urine.

Several bioanalytical methods have been developed to determine the etimicin concentration in different biological matrices of humans and animals by resonance Rayleigh scattering (Wang et al., 2010) or liquid chromatography-tandem mass spectrometry (LC-MS/MS) (Cui et al., 2014; Yao et al., 2017; Sun et al., 2022). Resonance Rayleigh scattering is a complex and time-consuming spectral method (Wang et al., 2010). As for the LC-MS/MS methods developed for preclinical studies and therapeutic drug monitoring, some used ion-pairing reagents in the mobile phase, leading to limit the lower limit of quantification (Cui et al., 2014); some adopted the antibiotics commonly used in clinical practice as the internal standard, limiting its application in human clinical studies (Yao et al., 2017); some was not with ideal chromatography retention and peak shape for etimicin (Sun et al., 2022). Besides, the quantitative ranges of etimicin have not been sufficiently wide in published LC-MS/MS methods. Hence, bioanalytical methods for the quantification of etimicin in human samples of serum and urine are lacking.

A dose-escalation phase-I clinical study was undertaken to clarify the pharmacokinetic characteristics of etimicin after administration of single and multiple doses, and to explore its breakpoint. In tandem with that study, an LC-MS/MS method was developed and validated for the determination of etimicin in human serum samples with a quantitative range sufficiently wide for the dose-escalation pharmacokinetic study. For the first time, we also developed and validated a quantitative LC-MS/MS method to determine the etimicin concentration in human urine samples. In order to avoid the shortcomings of the previous bioanalytical methods for etimicin, the LC-MS/MS platform was used without any ion-pairing agent in the mobile phase, or any clinical medication as the internal standard. These two simple, rapid, efficient, and robust methods were applied to the phase I clinical trial and we elucidated, for the first time, the comprehensive human pharmacokinetics of etimicin in humans.

## 2 Material and methods

### 2.1 Chemicals and reagents

Etimicin sulfate (90.7% purity), acting as the standard of etimicin, was purchased from National Institutes for Food and Drug Control (Beijing, China). The standard for carbamazepine-D_10_ (99% purity), acting as the internal standard (IS), was provided by Shanghai Zzbio (Shanghai, China).

HPLC-grade methanol and acetonitrile were obtained from Honeywell Burdick & Jackson (Morris Plains, NJ, USA). HPLC-grade acetic acid was purchased from Shanghai Aladdin Biochemical Technology (Shanghai, China). AR-grade ammonia solution and sodium hydroxide were sourced from Xilong Scientific (Shantou, China). Water was purified by the Milli-Q™ system from Millipore (Bedford, MA, USA). Blank human serum and blank human urine were collected from Chinese healthy volunteers (HVs) in Peking Union Medical College Hospital (Beijing, China) after written informed consent had been provided.

### 2.2 Instrumentation and analytical conditions

LC-MS/MS was done on an Acquity ultra-high-performance liquid chromatography system (Waters, Milford, MA, USA) coupled with a QTRAP 5500 mass spectrometer (Sciex, Toronto, ON, Canada) equipped with an electrospray ionization source operating in positive mode. Data were acquired and processed by Sciex Analyst™ 1.7.1.

An XTerra MS C18 column (2.1 × 150 mm, 3.5 μm; Waters) was used with water–ammonia solution–acetic acid (96:3.6:0.2, *v/v/v*) as mobile phase A and methanol as mobile phase B. Optimal peak shapes and retentions of analytes were achieved by isocratic elution (50% mobile phase B) at a flow rate of 0.35 ml/min with a column temperature of 40°C. The injection volume of each sample was 10 μL and the autosampler temperature was set to 10°C. The analytical time for each sample was 5 min.

MS parameters were optimized: gas 1, 55 psi; gas 2, 55 psi; curtain gas, nitrogen, 35 psi; collision gas, medium; temperature, 550°C. Compound-specific MS parameters for etimicin were optimized: declustering potential (DP), 180 V; entrance potential (EP), 8 V; collision energy (CE), 36 V; collision cell exit potential (CXP), 14 V. Compound-specific MS parameters for the IS were: DP, 100 V; EP, 8 V; CE, 29 V; CXP, 14 V. The following parent → product ion transitions were used for multiple-reaction monitoring: etimicin, *m/z* 478.280 → 191.110; IS, *m/z* 247.100 → 204.197; both had a dwell time of 150 ms.

### 2.3 Stock solutions, calibration standards, and quality control (QC) samples

Two sets of etimicin stock solution, both prepared with methanol–water (50/50, *v/v*) at 1.00 mg/ml, were used for the preparation of calibration standards and QC samples, respectively. A stock solution of the IS (1.00 mg/ml) was also prepared with methanol–water (50/50, *v/v*). These stock solutions were stored at −80°C.

For the preparation of calibration standards and QC samples, two series of working solutions of etimicin of suitable concentrations were prepared with methanol–water (50/50, *v/v*) by diluting the two sets of etimicin stock solution. Calibration standards in serum were prepared at 50.0, 100, 500, 1,000, 2500, 5000, 10,000, and 20,000 ng/ml by spiking the stock solution or working solutions of calibration standards in blank human serum. Similarly, calibration standards in urine were prepared by blank human urine at 50.0, 100, 250, 500, 1,000, 2500, 5000, and 10,000 ng/ml. Meanwhile, the lower limit of quantitation (LLOQ), low quality control (LQC), medium quality control (MQC), and high quality control (HQC) samples in human serum and human urine were prepared with stock solutions or working solutions of QC samples at 50.0, 150, 1,500, 15,000 ng/ml and 50.0, 150, 750, 7,500 ng/ml, respectively. Working solutions of the IS were prepared with methanol–water (50/50, *v/v*) at 40.0 ng/ml and 100 ng/ml for the pretreatment of urine samples and serum samples, respectively. The calibration standards and QC samples stated above were stored at −80°C, as were the two IS working solutions for urine (uISWS) preparation and serum (sISWS) preparation.

### 2.4 Sample preparation

Serum samples were prepared by protein precipitation. A serum sample (50 μL) was first mixed with sISWS (50 μL), and mixed with methanol (400 μL) subsequently to precipitate the proteins in the matrix. After vortex-mixing for 1 min, the mixture was centrifuged (17,000 × g, 10 min, room temperature). Then, the supernatant (200 μL) was mixed fully with mobile phase A (200 μL). This solution was diluted five-fold by mobile phase A–mobile phase B (50:50, *v/v*) before LC-MS/MS.

A urine sample (25 μL) was first mixed with uISWS (25 μL) and then with sodium hydroxide (50 μL) subsequently. After vortex-mixing, 900 μL of mobile phase A–mobile phase B (50:50, *v/v*) was added to the mixture, followed by vortex-mixing for 1 min. Then, the mixtures were centrifuged (17,000 × *g*, 10 min, room temperature). The supernatant was used for LC-MS/MS.

### 2.5 Method validation

We developed two LC-MS/MS bioanalytical methods for the determination of etimicin in human samples of serum and urine. Following the guidelines for the validation of bioanalytical methods set by China National Medical Products Administration (Chinese Pharmacopoeia Commission, 2020), US Food and Drug Administration (US Food and Drug Administration, 2018), and European Medicines Agency (European Medicines Agency, 2011), these two methods were validated comprehensively including accuracy, precision, linearity, selectivity, matrix effect, recovery, dilution integrity, stability, and carry-over. For each analytical batch of serum samples or urine samples, dual eight-point calibration curves, ranging from 50.0 ng/ml to 20,000 ng/ml for serum samples and 50.0 ng/ml to 10,000 ng/ml for urine samples, were analyzed to quantify the analyte.

#### 2.5.1 Selectivity

Six lots of blank serum samples and urine samples were collected from six Chinese HVs. All free from analyte or the IS, they were prepared to assess method selectivity. The peak area of a potential interfering peak from the biological matrix with a retention time similar to etimicin or the IS had to be <20% and <5% that of etimicin and the IS in LLOQ samples, respectively.

#### 2.5.2 Linearity

The linearity of the two methods was assessed over a concentration range of 50.0–20000 ng/ml for serum samples and 50.0–10000 ng/ml for urine samples, respectively. Each analytical batch contained two sets of calibration curves. Linearity was assessed by least square linear regression analysis between the peak area ratios of the analyte to the IS (y) and nominal concentrations (x) with a weighting factor of 1/x^2^. For each analytical batch, the percent relative error (%RE) had to be within ±15% of the nominal concentrations for ≥75% of the calibration standards except LLOQs, of which the %RE had to be within ±20%.

#### 2.5.3 Accuracy and precision

Three consecutive batches, each containing QC samples (*n* = 6) at the four concentrations (LLOQ, LQC, MQC, HQC), were analyzed on 2 days by two analysts to evaluate the intra- and inter-batch accuracy and precision of the two LC-MS/MS methods for etimicin in human samples of serum and urine. The QC concentrations of etimicin were 50.0, 150, 1,500, and 15,000 ng/ml for serum samples, and 50.0, 150, 750, and 7,500 ng/ml for urine samples. The intra- and inter-batch accuracy (%RE) had to be less than ±15% for LQC, MQC, HQC, and less than ±20% for LLOQ. The intra- and inter-batch precision, expressed as the percent relative standard deviation (%RSD), had to be ≤ 15% for LQC, MQC, HQC, and less than 20% for LLOQ.

#### 2.5.4 Matrix effect and recovery

For serum samples and urine samples, the matrix effect and recovery were evaluated in the same batch respectively, which contained six replicates of neat solutions of the analyte and the IS, post-extraction spiked QC samples from six HVs, and six replicates of QC samples. Neat solutions of the analyte and IS were prepared with mobile phase A–mobile phase B (50:50, *v/v*) at identical concentrations to those of post-preparation QC samples. Meanwhile, blank matrix extracts originating from six HVs were prepared and spiked with solutions of the analyte and IS to achieve post-extraction spiked QC samples with concentrations equal to post-preparation QC samples.

The matrix effect of serum samples and urine samples was evaluated by the matrix factor (MF). The MF was calculated by the peak area ratios of the analyte or IS in the presence and absence of the matrix. The acceptance criterion of the matrix effect was that the %RSD of the IS normalized MF (calculated by dividing the MF of the analyte with the MF of IS) for the six sources of matrix should be ≤ 15%.

The recovery of etimicin and IS in serum samples and urine samples was assessed by the peak area ratios of extracted QC samples and post-extraction spiked QC samples. The %RSD of the recovery across the three QC concentrations had to be ≤ 20%.

#### 2.5.5 Dilution integrity

To assess the dilution integrity, a dilution quality control (DQC) sample of serum was prepared at 75,000 ng/ml in blank human serum with a dilution factor of 10. For urine samples, DQC was prepared at 400,000 ng/ml with blank human urine, of which the dilution factors were 100 and 500. DQC samples were first diluted with the corresponding dilution factor by the corresponding blank matrix before sample preparation. Then, diluted DQC samples were quantified to evaluate the dilution integrity of serum samples and urine samples. For serum samples and urine samples, the average %RE of the back-calculated concentration of DQC had to be within ±15% of the nominal concentration with a %RSD ≤15%.

#### 2.5.6 Stability

The stability of etimicin in human serum samples was assessed by analyzing six replicates of serum LQC samples and serum HQC samples after storage under certain conditions. After storage at 25°C for 18 h, serum QC samples were assessed for short-term stability. Long-term stability was assessed after storage of serum QC samples for 60 days at −30°C and −80°C, respectively. As for freeze-thaw stabilities of serum samples, serum QC samples were analyzed after four freeze–thaw cycles from −80°C to 25°C. Also, processed serum QC samples were stored at 10°C for 72 h before being quantified against freshly prepared calibration standards to evaluate the processed stability.

Similarly, the stability of etimicin in human urine samples was evaluated by analyzing six replicates of urine LQC samples and urine HQC samples after storage under certain conditions: short-term stability following storage at 25°C for 24 h; long-term stability following storage at −80°C and −30°C for 120 days; freeze–thaw stability after five freeze–thaw cycles from −80°C to 25°C; processed stability after storage at 10°C for 68 h.

For each assessment of stability, the analyte was considered to be stable if the average value of the QC replicates was within ±15% of the nominal concentration with a %RSD ≤15%.

#### 2.5.7 Carry-over

In each validation batch for serum samples or urine samples, two double-blank samples (without etimicin or IS) were analyzed directly after the upper limit of quantification (ULOQ) samples had been run to determine the carry-over effect. The peak area of any carry-over peak had to be ≤ 20% and ≤5% that of etimicin and the IS in LLOQ samples, respectively.

### 2.6 Pharmacokinetics study in Chinese HVs

A phase I clinical study of etimicin was conducted in Peking Union Medical College Hospital. The study protocol was approved (EC# I-22PJ127) by the ethics committee of Peking Union Medical College Hospital and conducted in compliance with the Declaration of Helsinki 1964 and its later amendments. The study cohort was Chinese HVs, aiming to investigate the comprehensive pharmacokinetics and breakpoint of etimicin.

This was an open-label, dose-escalation, phase I study, in which subjects received single and multiple doses of etimicin by intravenous infusion for 1.0 h. Eligible individuals were enrolled and assigned to four groups (A–D). HVs in groups A, B, and C were administered a single dose of etimicin (120, 200, and 300 mg, respectively) on day-1. Group D was used to explore the pharmacokinetic characteristics of etimicin after administration of multiple doses. Individuals in group D received etimicin (150 mg) once a day on day-1, received etimicin (150 mg) twice a day at a time interval of 12 h from day-2 to day-5, and received etimicin (150 mg) once a day on day-6.

For single-dose groups, blood samples were collected from a peripheral vein pre-dose as well as 0.5 (except group A), 1, 1.25, 1.5, 2, 2.5, 3, 4, 5, 6, 8, 10, 12, and 24 h after dosing. For the multiple-dose group, blood samples were collected at certain time points on day-1 and day-6 (pre-dose as well as 1, 1.25, 1.5, 2, 2.5, 3, 4, 5, 6, 8, 10, 12, and 24 h after dosing) as well as pre-dose on day-4 and day-5. Urine samples of single-dose groups were collected pre-dose as well as 0–2, 2–4, 4–8, 8–12, and 12–24 h after dosing.

Blood samples were stored at room temperature for 60 min initially, and then centrifuged (2000 × *g*, 10 min, 4°C) to obtain serum samples. The acquired serum samples were frozen at −80°C before LC-MS/MS analysis. Urine samples were also stored at −80°C until LC-MS/MS analysis.

The pharmacokinetic parameters of etimicin were calculated through non-compartment analysis by Phoenix WinNonlin 8.3.1 (Certara, Princeton, NJ, USA).

In addition, incurred sample reanalysis (ISR) was conducted during the analysis of clinical serum samples and urine samples. For the ISR assessment, more than 10% of the clinical samples should be chosen and they should be reanalyzed according to fresh calibration curves. The time point of the ISR samples should be close to the time point of peak concentration or near the end of the terminal phase of the pharmacokinetic profile. The ISR concentration should be within ±20% of the mean of original concentration and ISR concentration. This requirement should be met by at least three-thirds of the ISR samples.

## 3 Results and discussion

### 3.1 Development and optimization of the methods

**FIGURE 1:**
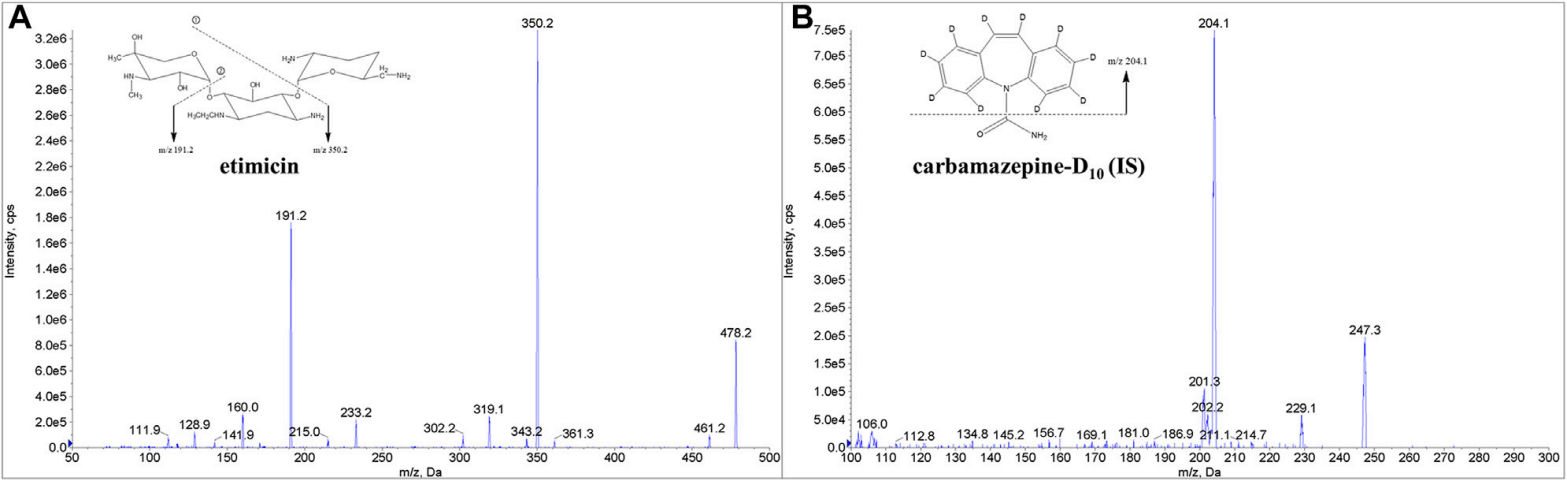
Chemical structures and mass spectra of etimicin **(A)** and the IS **(B)**.

The two methods were developed for the quantitative determination of etimicin in human samples of serum or urine by LC-MS/MS. First, MS parameters were optimized with solutions of etimicin and the IS at 500 ng/ml in water–methanol (50:50, *v/v*), respectively. To achieve optimal sensitivity, a series of MS parameters were tuned in positive mode: desolvation temperature, gas settings, DP, EP, CE, and CXP. Typical product ion mass spectra for etimicin and IS are shown in Figure 1.

**FIGURE 2:**
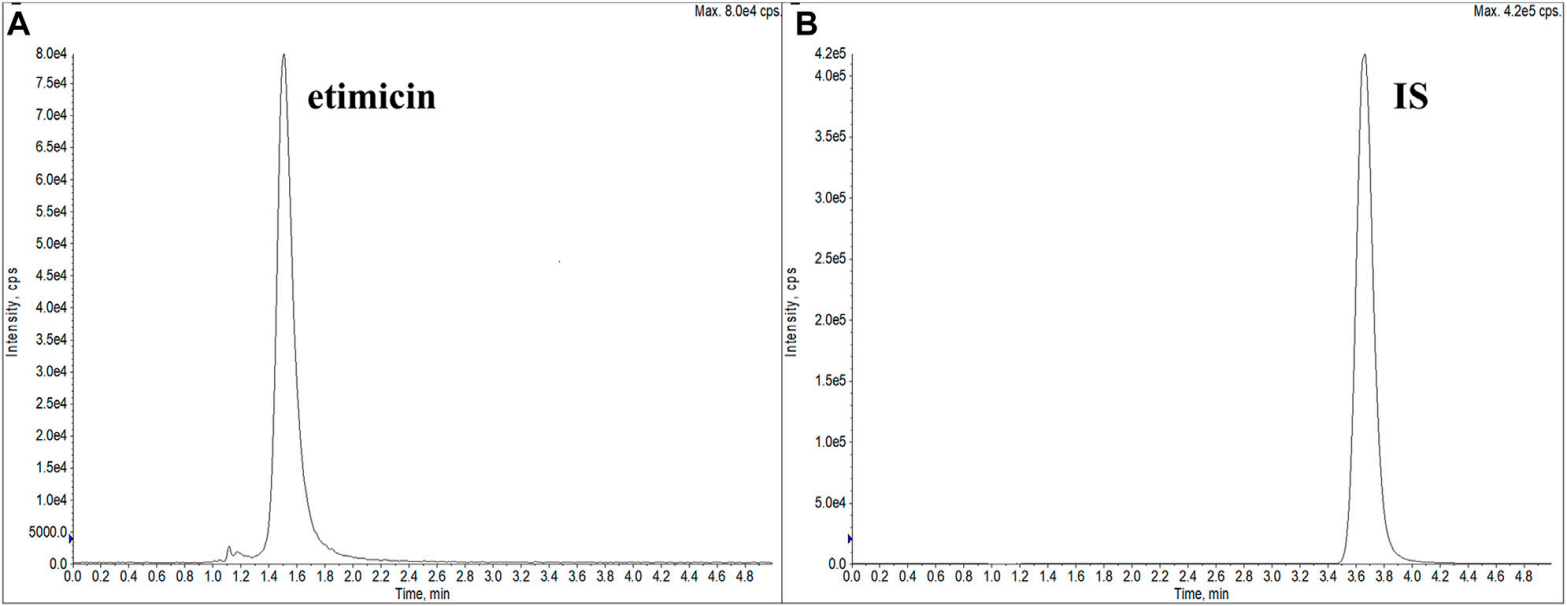
Typical extracted ion chromatograms of etimicin **(A)** and the IS **(B)** (retention time: etimicin = 1.5 min, IS = 3.7 min).

Different types of columns from Waters were explored: Acquity UPLC BEH C18 (2.1 × 50 mm, 1.7 μm), Acquity UPLC CSH C18 (2.1 × 50 mm, 1.7 μm), Acquity UPLC BEH HILIC (2.1 × 50 mm, 1.7 μm), and XTerra MS C18 (2.1 × 150 mm, 3.5 μm). We also investigated modifiers of the mobile phase: formic acid, acetic acid, and ammonia solution. Finally, optimal peak separations and peak shapes were achieved on the Waters XTerra MS C18 column (2.1 × 150 mm, 3.5 μm) with water–ammonia solution–acetic acid (96:3.6:0.2, *v/v/v*) as mobile phase A and methanol as mobile phase B. Ion-pairing agents were not used in the mobile phases, which ensured that the LLOQ could be sufficiently low. Under isocratic elution of 50% mobile phase B, the typical retention time of etimicin and the IS was 1.5 min and 3.7 min, respectively (Figure 2).

Different sample-preparation methods were explored to reduce interference from the matrix: protein precipitation, liquid–liquid extraction, and solid-phase extraction. In consideration of succinctness and rapidity, protein precipitation was finally adopted for serum samples. Methanol was the optimal solvent for protein precipitation, and had a good recovery and only a minimal matrix effect. Besides, considering that the methods were developed for a human pharmacokinetic study, a deuterated reagent was adopted as the IS to remove endogenous interference introduced by the sample-preparation process. A deuterated version of etimicin is not available commercially, so we chose carbamazepine-D_10_ as the IS. The retention time and ionization of the IS were not similar to those of etimicin, but the matrix effect and recovery of the IS and etimicin were comparable.

### 3.2 Method validation

#### 3.2.1 Selectivity

**FIGURE 3:**
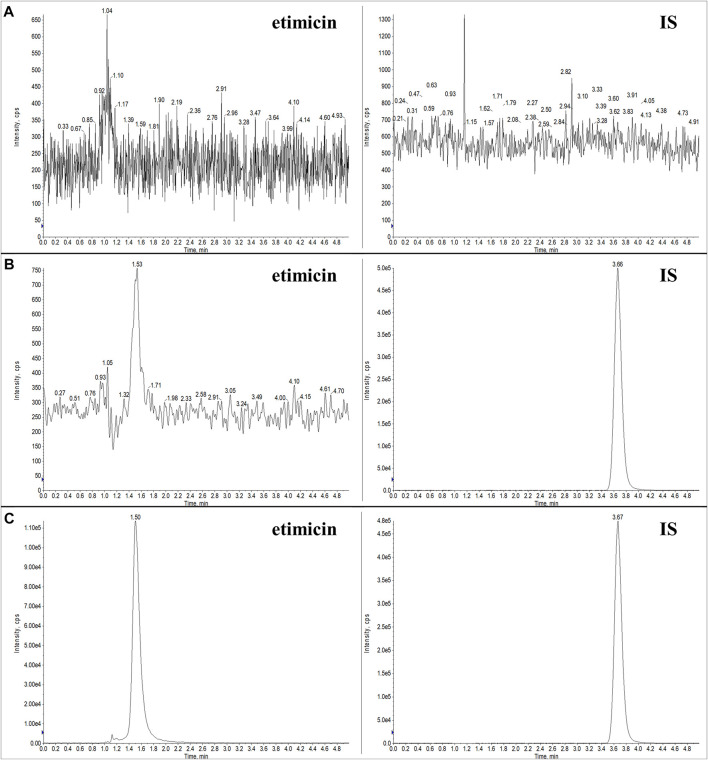
Representative LC-MS/MS chromatograms of etimicin and the IS in blank serum **(A)**, LLOQ serum **(B)**, and serum sample from Chinese HVs after administration of etimicin **(C)**. Transitions monitored for quantitation were *m/z* 478.280 → 191.110 for etimicin and *m/z* 247.100 → 204.197 for the IS, respectively.

**FIGURE 4:**
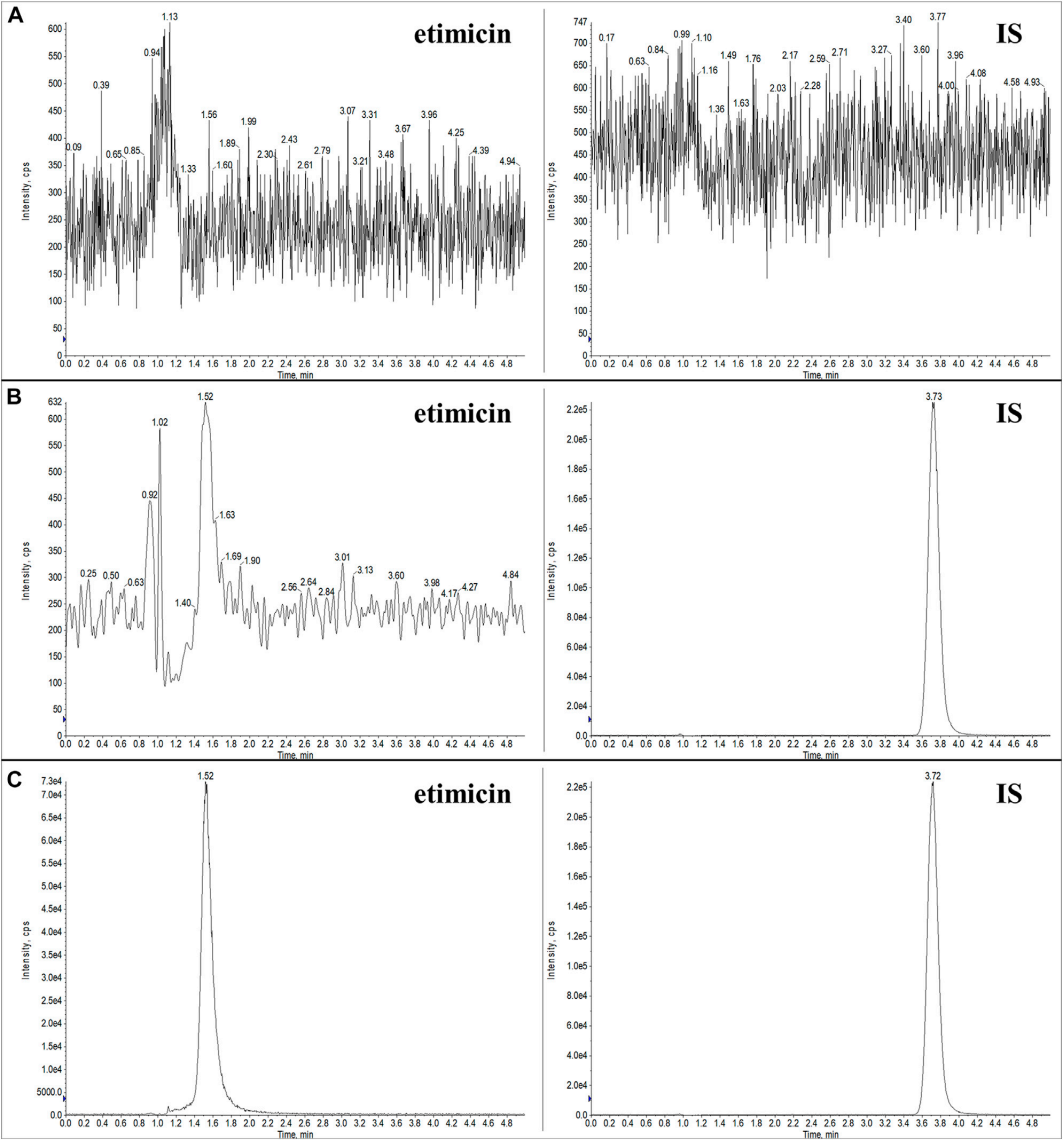
Representative LC-MS/MS chromatograms of etimicin and the IS in blank urine **(A)**, LLOQ urine **(B)**, and urine sample from Chinese HVs after administration of etimicin **(C)**. Transitions monitored for quantitation were *m/z* 478.280 → 191.110 for etimicin and *m/z* 247.100 → 204.197 for the IS, respectively.

Typical chromatograms of blank serum, LLOQ serum sample, and human serum sample (1.25 h after etimicin administration for HV #3004) are shown in Figure 3. Typical chromatograms of blank urine, LLOQ urine sample, and human urine sample (0–2 h after etimicin administration for HV #3001) are shown in Figure 4. The typical retention time of etimicin and the IS was 1.5 min and 3.7 min, respectively. Endogenous interference was not observed at the retention time of the analyte or IS in serum samples or urine samples.

#### 3.2.2 Linearity

The calibration range of etimicin was 50.0–20000 ng/ml and 50.0–10000 ng/ml for serum samples and urine samples, respectively. For serum samples and urine samples, the calibration curves showed good linearity in each validation batch, all with a regression coefficient (R^2^) >0.99.

#### 3.2.3 Accuracy and precision

The intra- and inter-batch accuracy and precision of the two methods for serum samples and urine samples are shown in Table 1. For serum samples, the intra- and inter-batch accuracy (%RE) ranged from −12.7% to 8.7% across LQC, MQC, and HQC and between −10.2% to 6.4% for LLOQ. The intra- and inter-batch precision (%RSD) ranged from 1.5% to 9.5% across LQC, MQC, and HQC and between 7.2% to 13.2% for LLOQ. For urine samples, the intra- and inter-batch accuracy (%RE) ranged from −5.3% to 10.8% across LQC, MQC, and HQC and between −6.0% to 0.2% for LLOQ. The intra- and inter-batch precision (%RSD) ranged from 2.6% to 7.7% across LQC, MQC, and HQC and between 9.3% to 10.8% for LLOQ. The intra- and inter-batch accuracy and precision of etimicin in serum samples and urine samples met the acceptance criteria.

**TABLE 1:** Inter- and intra-batch accuracy and precision of etimicin in human samples of serum and urine.

	Serum QC concentration (ng/ml)				Urine QC concentration (ng/ml)			
	LLOQ	LQC	MQC	HQC	LLOQ	LQC	MQC	HQC
	50	150	1,500	15,000	50	150	750	7,500
Batch 1 (*n* = 6)								
Mean (ng/ml)	46.1	147	1,470	13,700	50.1	157	724	7,190
RSD (%)	13.2	3.2	2.4	2.6	10.8	6.4	6.5	6.4
RE (%)	−7.8	−2.0	−2.0	−8.7	0.2	4.7	−3.5	−4.1
Batch 2 (*n* = 6)								
Mean (ng/ml)	44.9	131	1,350	14,500	47.0	142	713	8,230
RSD (%)	8.4	2.8	3.0	1.5	9.7	4.7	6.6	2.6
RE (%)	−10.2	−12.7	−10.0	−3.3	−6.0	−5.3	−4.9	9.7
Batch 3 (*n* = 6)								
Mean (ng/ml)	53.2	163	1,580	14,600	47.8	154	770	8,310
RSD (%)	7.2	3.8	2.6	1.5	9.3	4.2	5.7	3.0
RE (%)	6.4	8.7	5.3	−2.7	−4.4	2.7	2.7	10.8
Inter-batch (*n* = 18)								
Mean (ng/ml)	48.1	147	1,470	14,300	48.2	151	736	7,910
RSD (%)	12.1	9.5	7.1	3.5	9.7	6.6	6.8	7.7
RE (%)	−3.8	−2.0	−2.0	−4.7	−3.6	0.7	−1.9	5.5

#### 3.2.4 Matrix effect and recovery

The matrix effect of etimicin in serum samples, normalized by the IS, was 84.0–91.1% with a %RSD <3.6%. For urine samples, the IS-normalized matrix effect was 82.8–93.3% with a %RSD <13.8%. These results indicated that the matrix effects of etimicin were slight and consistent for our methods. The recovery from serum samples was 89.5%, 81.0%, and 79.0% for etimicin at LQC, MQC, and HQC, and was 97.0% for the IS across LQC, MQC, and HQC. The recovery from urine samples was 104.7%, 105.6%, and 101.4% for etimicin at LQC, MQC, and HQC, and was 101.7% for the IS across LQC, MQC, and HQC. The recovery of etimicin remained constant across the quantitative range for serum samples and urine samples.

#### 3.2.5 Dilution integrity

Six replicates of serum DQC (75,000 ng/ml) were diluted 10-fold with pooled blank human serum before sample preparation, and the %RE and %RSD were −6.3% and 1.4%, respectively. Two dilution factors (100 and 500) were evaluated with urine DQC (400,000 ng/ml). The accuracy (%RE) and precision (%RSD) of urine DQC were 0.2% and 6.4%, respectively, with a dilution factor of 100, and were 96.7% and 1.9%, respectively, with a dilution factor of 500. Therefore, one should dilute serum samples with concentrations above the ULOQ by 10-fold with mixed blank serum before sample preparation. It was advisable to dilute urine samples with concentrations above the ULOQ by 100-fold or 500-fold with mixed blank urine before sample preparation.

#### 3.2.6 Stability

The stability of etimicin in serum samples was assessed from the period of storage to analysis (Table 2). Etimicin showed good stability in serum after storage at −80°C or −30°C for 60 days. Etimicin was stable in serum after storage at 25°C for 18 h or after four freeze–thaw cycles from −80°C to 25°C. Etimicin was stable in processed serum samples for 72 h at 10°C after sample preparation.

**TABLE 2:** Short-term stability, long-term stability, freeze–thaw stability, and processed stability of etimicin in human serum samples.

	LQC 150 ng/ml	HQC 15000 ng/ml
Short-term stability (25°C, 18 h) (*n* = 6)		
Mean (ng/ml)	145	13,900
RSD (%)	3.2	1.8
RE (%)	−3.7	−7.6
Long-term stability (−80°C, 60 days) (*n* = 6)		
Mean (ng/ml)	151	16,800
RSD (%)	2.3	3.5
RE (%)	0.7	11.7
Long-term stability (−30°C, 60 days) (*n* = 6)		
Mean (ng/ml)	152	16,700
RSD (%)	9.1	1.5
RE (%)	1.6	11.4
Freeze–thaw stability (−80°C to 25°C, four cycles) (*n* = 6)		
Mean (ng/ml)	154	15,500
RSD (%)	3.7	5.3
RE (%)	2.4	3.4
Stability of processed samples (10°C, 72 h) (*n* = 6)		
Mean (ng/ml)	155	14,200
RSD (%)	7.3	3.1
RE (%)	3.1	−5.1

The stability of etimicin in urine was evaluated in a similar manner (Table 3 **)**. Etimicin showed good stability in urine after storage at −80°C and −30°C for 120 days. It remained stable in urine after storage at 25°C for 24 h or after five freeze–thaw cycles from −80°C to 25 °C. Etimicin was stable in processed urine samples for 68 h at 10°C after sample preparation.

**TABLE 3:** Short-term stability, long-term stability, freeze–thaw stability, and processed stability of etimicin in human urine samples.

	LQC 150 ng/ml	HQC 7500 ng/ml
Short-term stability (25°C, 24 h) (*n* = 6)		
Mean (ng/ml)	132	7,910
RSD (%)	7.3	6.0
RE (%)	−12.1	5.5
Long-term stability (−30°C, 120 days) (*n* = 6)		
Mean (ng/ml)	142	7,580
RSD (%)	9.3	2.9
RE (%)	-5.3	1.1
Long-term stability (−80°C, 120 days) (*n* = 6)		
Mean (ng/ml)	133	7,130
RSD (%)	7.1	2.5
RE (%)	−11.3	−4.9
Freeze–thaw stability (−80°C to 25°C, five cycles) (*n* = 6)		
Mean (ng/ml)	160	8,310
RSD (%)	6.7	7.9
RE (%)	6.3	10.8
Stability of processed samples (10°C, 68 h) (*n* = 6)		
Mean (ng/ml)	148	7,650
RSD (%)	11.3	7.3
RE (%)	−1.4	2.0

#### 3.2.7 Carry-over

For serum samples and urine samples, a significant chromatographic peak was not observed in the double-blank sample after the ULOQ sample had been run in each batch. Hence, the carry-over effects for both methods were negligible.

### 3.3 Application of the two methods in a pharmacokinetic study

The two validated LC-MS/MS methods were applied to a phase I pharmacokinetic study of etimicin in Chinese HVs. Currently, six HVs have been enrolled in each group of this trial.

**FIGURE 5:**
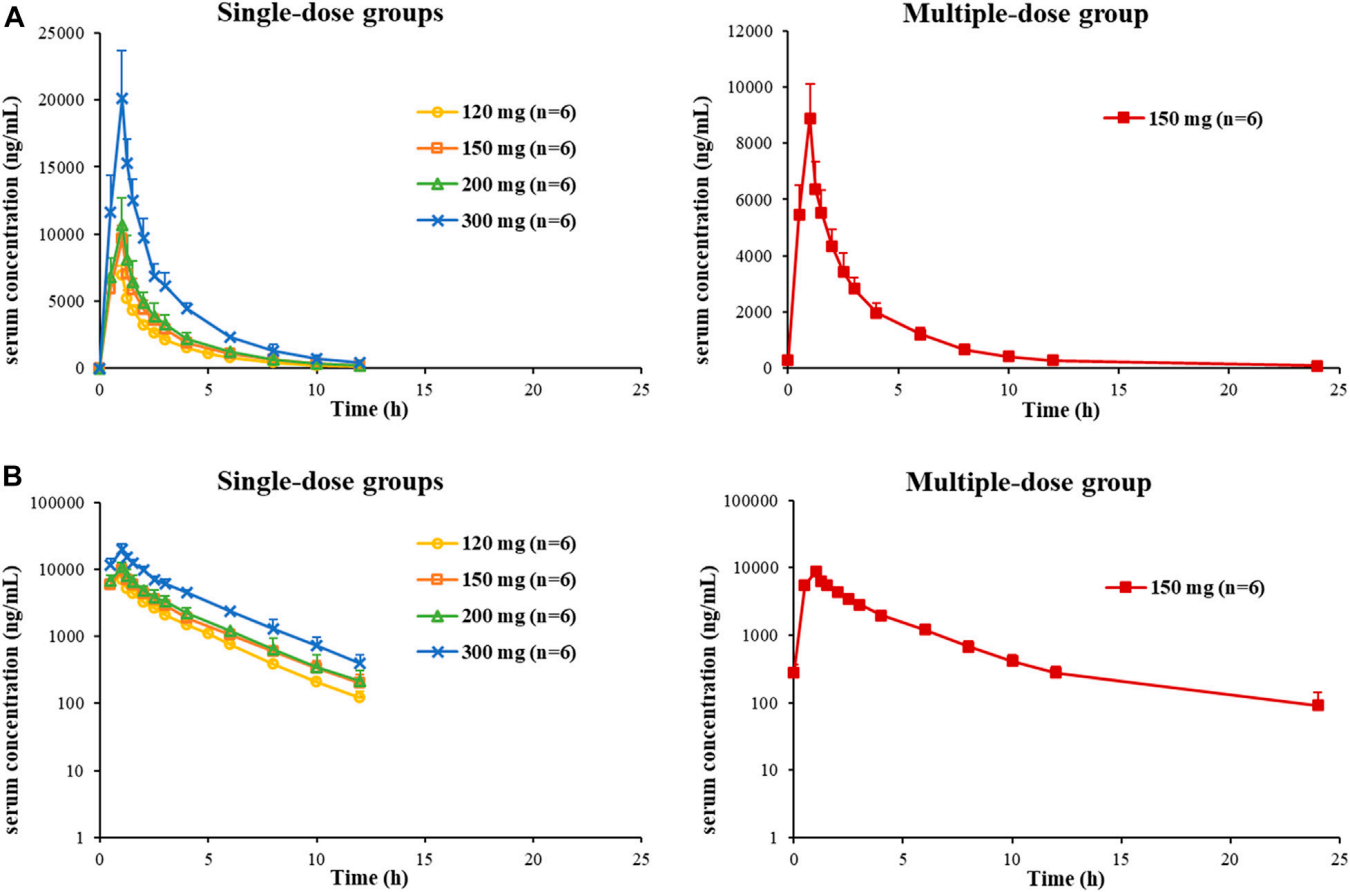
Average serum concentration–time curves of etimicin in Chinese HVs after a single dose and multiple doses, shown in Cartesian coordinates **(A)** and semilogarithmic coordinates **(B)**.

**FIGURE 6:**
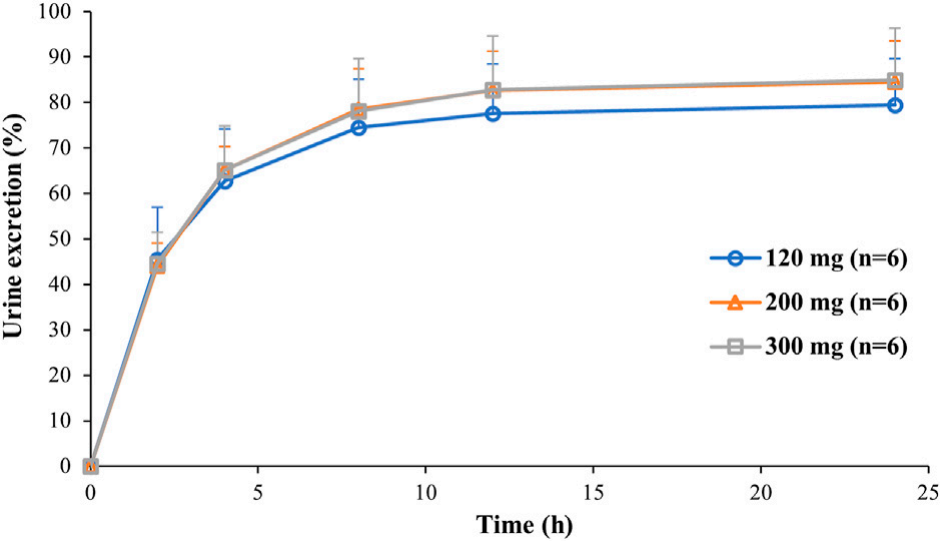
Average urinary excretion–time curves of etimicin after intravenous administration of etimicin (120, 200, 300 mg) in Chinese HVs.

So far, 444 serum samples and 108 urine samples have been analyzed. The average serum concentration–time curves of etimicin in Chinese HVs after a single dose and multiple doses are shown in Figure 5. The pharmacokinetic parameters of etimicin for the same dose groups are shown in Table 4. The maximum serum concentrations of etimicin after a single dose were 6,910–20200 ng/ml at a dose range of 100–300 mg, and were observed at the end of the 1-h intravenous infusion. The half-life and clearance of etimicin in serum were 2.56–2.89 h and 5.80–7.50 L/h after a single dose, respectively, and were approximately consistent in a dose range of 100–300 mg. Obvious accumulation was not observed after multiple doses of etimicin at 150 mg. The half-life and clearance of etimicin were 5.16 h and 5.90 L/h, respectively, after administration of multiple doses. The analysis of etimicin excretion in urine is shown in Figure 6. According to the urinary-excretion profile, 79.4–84.9% of the etimicin dose was eliminated through urine 24 h after a single dose of etimicin.

**TABLE 4:** Pharmacokinetic parameters (mean (SD), *n* = 6) of etimicin in Chinese HVs after a single dose (120 mg, 150 mg, 200 mg, 300 mg) and multiple doses (150 mg).

	Single-dose groups				Multiple-dose group
Parameter^a^	**120 mg**	**150 mg**	**200 mg**	**300 mg**	**150 mg**
T_max_ (h)	1.0 (0)	1.0 (0)	1.0 (0)	1.0 (0)	1.0 (0)
C_max_ (ng/ml)	6,910 (722)	9,680 (919)	10,700 (2020)	20,200 (3,520)	8,890 (1,209)
AUC_last_ (h^a^ng/mL)	16,941 (1182.5)	23,598 (2791.0)	26,553 (5400.5)	51,065 (6191.3)	25,281 (4634.6)
AUC_%Extrap_obs (%)	2.2 (0.59)	3.1 (1.43)	3.4 (2.48)	2.2 (1.04)	3.5 (2.94)
t_1/2_ (h)	2.89 (1.570)	2.57 (0.539)	2.77 (1.018)	2.56 (0.704)	5.16 (3.181)
K_e_ (1/h)	0.28 (0.085)	0.28 (0.060)	0.27 (0.070)	0.29 (0.067)	0.18 (0.093)
AUCINF_obs (h^a^ng/mL)	17,326 (1163.2)	24,378 (3060.1)	27,456 (5309.6)	52,203 (5971.7)	26,205 (4793.4)
Cl (L/h)	6.95 (0.497)	6.24 (0.818)	7.50 (1.366)	5.80 (0.591)	5.90 (1.172)
Vz (L)	28.78 (14.967)	22.82 (4.238)	30.70 (15.217)	21.55 (6.429)	40.98 (20.472)
Fe (%)	79.4 (10.29)	NA^b^	84.5 (8.98)	84.9 (11.43)	NA^b^

^a^ T_max_, Time of maximum observed concentration; C_max_, Maximum observed concentration, occurring at T_max_; AUC_last_, Area under the curve from the time of dosing to the last measurable (positive) concentration; AUC_%Extrap_obs, Percentage of AUCINF_obs due to extrapolation from the time of the last observation to infinity; t_1/2_, Terminal half-life; K_e_, First order rate constant associated with the terminal (log-linear) portion of the curve; AUCINF_obs, Area under the curve from the time of dosing extrapolated to infinity, based on the last observed concentration; Cl, total body clearance; Vz, Volume of distribution based on the terminal phase; Fe, The fraction excreted.

^b^ NA, not applicable.

The bold values were the dose group names for the single- and multiple-dose pharmacokinetic research.

Additionally, 12.6% and 29.6% of the clinical serum samples and clinical urine samples were selected for the ISR assessment, with respective passing rates of 82.1% and 80.6%. The ISR assessments for these two bioanalysis methods both met the acceptance criterion.

Several pharmacokinetic studies using a single dose of etimicin have been reported (Zhang and Yin, 1999; Yin et al., 2000; Wang et al., 2002; Ouyang, 2007). In those studies, pharmacokinetics was evaluated in only one single-dose group (150 mg or 200 mg) and drug concentrations were measured by micro-bioassays. The single-dose or multiple-dose pharmacokinetics of etimicin in a certain dose-escalation range have not been clarified before. Based on the two reliable and rapid LC-MS/MS methods developed in this paper, we captured the detailed and comprehensive human pharmacokinetic profile of etimicin for the first time, including the single-dose pharmacokinetics in 120 mg-300 mg and the multiple-dose pharmacokinetics. These pharmacokinetic parameters will be further used in the exploration of the breakpoint in Chinese by Monte Carlo simulation, which will advance the rational use of antibiotics in clinical practice.

## 4 Conclusion

We developed and validated two rapid and reliable LC-MS/MS methods for the quantification of etimicin in human samples of serum and urine, which was the first bioanalytical method for the determination of etimicin in human urine samples by LC-MS/MS. Antibiotics employed commonly in clinical practice or ion-pairing agents were not used as the IS or mobile phase modifier in these two methods. The two methods were both with acceptable sensitivity and wide quantitative ranges. These two methods showed good linearity, selectivity, accuracy, and precision across a range of 50.0–20000 ng/ml for serum samples and 50.0–10000 ng/ml for urine samples, respectively. And they were also found to have consistent recoveries over a linear range and slight matrix effects. These two high-throughput quantification methods were applied successfully to study the pharmacokinetics of etimicin in a dose-escalation, phase I clinical trial with Chinese HVs. Based on these two methods we ascertained, for the first time, the comprehensive pharmacokinetics of etimicin in humans. The pharmacokinetic parameters we determined will be used for the exploration of the breakpoint in Chinese HVs by Monte Carlo simulation, which could be used to advance the rational use of antibiotics in clinical practice.

## Data Availability

The original contributions presented in the study are included in the article/Supplementary Material, further inquiries can be directed to the corresponding author.
